# Tamoxifen-induced neutropenia and leukopenia in a breast cancer patient: a case report and literature review

**DOI:** 10.3389/fonc.2026.1691480

**Published:** 2026-04-01

**Authors:** Cheng Liu, Fadong Xu, Qinglai Liu

**Affiliations:** Department of Breast Surgery, Liaocheng Maternity and Child Health Care Hospital, Liaocheng, Shandong, China

**Keywords:** adverse drug reaction, agranulocytosis, breast neoplasm cancer, endocrine therapy, leukopenia, tamoxifen

## Abstract

**Background:**

Tamoxifen is a cornerstone of endocrine therapy for hormone receptor-positive breast cancer. While its common side effects are well-documented, severe hematological toxicities such as neutropenia and leukopenia are extremely rare.

**Case presentation:**

We report a case of a 37-year-old premenopausal woman with hormone receptor-positive breast cancer who developed neutropenia and leukopenia three months after initiating adjuvant tamoxifen (20 mg/day). Despite transient improvements with supportive therapies (leucogen tablets and Compound Ejiao Jiang), cytopenias persisted. Tamoxifen was discontinued and switched to ovarian function suppression (leuprolide) plus an aromatase inhibitor (exemestane). Within four weeks, neutrophil and leukocyte counts normalized and remained stable over six months of follow-up.

**Conclusion:**

This case highlights the importance of recognizing tamoxifen-induced hematological toxicity. Regular blood count monitoring is essential, and in premenopausal women intolerant to tamoxifen, switching to ovarian function suppression plus an aromatase inhibitor is a safe and effective alternative.

## Introduction

**Figure 1:**
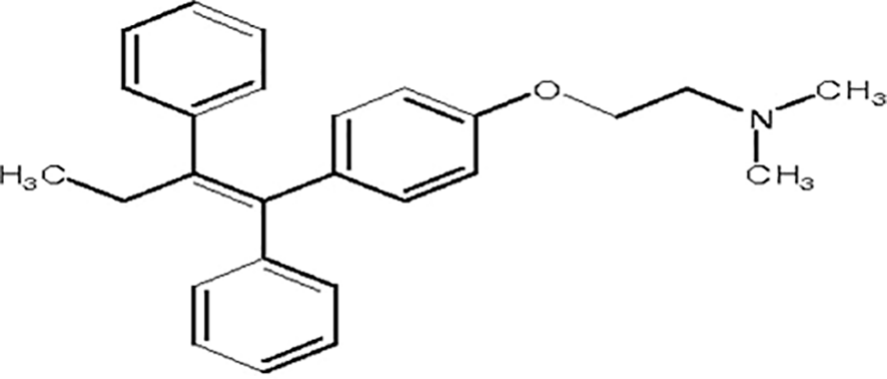
Chemical structure of tamoxifen [(Z)-2-[4-(1,2-diphenylbut-1-enyl)phenoxy]-N,N-dimethylethanamine].

Tamoxifen (TAM), a non-steroidal anti-estrogen drug (**Figure 1**), is widely used in the adjuvant endocrine therapy for hormone receptor (HR)-positive breast cancer, demonstrating efficacy in reducing patient mortality and recurrence rates (1). It acts as a competitive antagonist of estradiol, binding to estrogen receptors in breast cells without significant transcriptional activation. Tamoxifen upregulates the production of transforming growth factor-beta (TGF-β) and specifically inhibits protein kinase C, both of which contribute to the suppression of estrogen dependent tumor cells. The primary side effects of tamoxifen are related to its anti-estrogenic actions and include menopausal symptoms such as menstrual disorders, hot flashes, vaginal bleeding, venous thrombosis, arthralgia, and bone loss (2). Hematological toxicity, including leukopenia and thrombocytopenia, occurs occasionally, with agranulocytosis being exceptionally rare and scarcely documented in the literature.

The molecular structure of tamoxifen [(Z)-2-[4-(1,2-diphenylbut-1-enyl)phenoxy]-N,N-dimethylethanamine] comprises a triphenylethylene core and a basic aminoethoxy side chain (**Figure 1**). The Z-isomer optimally fits within the estrogen receptor ligand-binding domain, displacing helix 12 and blocking coactivator recruitment—the structural basis for its antagonist activity (22, 23).

Binding affinity derives from three key molecular interactions: (1) hydrogen bonding between the phenolic ring and Glu353/Arg394; (2) hydrophobic contacts from the triphenylethylene core; and (3) ionic interactions of the basic side chain with Asp351 (24). These same structural features that enable precise receptor targeting may also contribute to rare off-target effects, including potential interactions with hematopoietic progenitors expressing variant estrogen receptors (12, 13).

Herein, we report a case of tamoxifen-induced agranulocytosis and leukopenia. By adjusting the treatment regimen, the hematological toxicity was successfully managed. This case aims to enhance clinical awareness of this rare adverse reaction and provide insights for its diagnosis and management.

## Case data

A 37-year-old female patient was diagnosed with right breast invasive ductal carcinoma (histological Grade II). Immunohistochemistry results were as follows: Estrogen Receptor (ER) positive, Progesterone Receptor (PR) positive, Human Epidermal Growth Factor Receptor 2 (HER2) (IHC 2+ with no amplification by FISH), and a proliferation index Ki-67 of approximately 15%. In September 2022, she underwent a right total mastectomy and sentinel lymph node biopsy. The final tumor TNM stage was pT1N0M0. She declined Oncotype DX testing to evaluate potential benefit from chemotherapy due to personal reasons. She subsequently received four cycles of TC chemotherapy (docetaxel 75 mg/m² and cyclophosphamide 500 mg/m² every 3 weeks). According to the 2022 Chinese guidelines for the diagnosis and treatment of breast cancer, patients have histological grade II risk factors, and ovarian function inhibitors (OFS) combined with tamoxifen adjuvant endocrine therapy should be preferred after chemotherapy (3). However, the patient only received tamoxifen (20 mg/day) alone for financial reasons.

## Treatment course

At baseline, the patient was in good general condition, with no palpable lymphadenopathy, no skin changes, and a well-healed surgical scar. Systemic examination was unremarkable. During follow-up visits, she reported fatigue but no fever, chills, or signs of infection. Physical examinations remained normal throughout, with no hepatosplenomegaly or mucosal ulcerations.

Three months after starting tamoxifen (March 2023), blood routine showed leukopenia (white blood cell [WBC]count 3.00 × 10/L, grade 1) and neutropenia (absolute neutrophil count 1.39 × 10/L, grade 2). Platelets and hemoglobin were normal. She denied any new medications, infections, or nutritional deficiencies. Given the rarity of mild hematological toxicities (grade 1-2) and tamoxifen-related hematological toxicities, tamoxifen was continued under close monitoring at the original dose, supplemented by oral leucogen tablets (20 mg three times daily) to promote leukocytosis and neutrophil recovery. The patient had the first follow-up visit in March 2023 (3 months after tamoxifen treatment) with white blood cell count (WBC) 3.00 × 10 ^/L (grade 1 toxicity) and neutrophil count 1.39 × 10 ^/L (grade 2 toxicity). The initial dose of tamoxifen was continued and leucogen tablets (20 mg tid) was added. One month later (April 2023), WBC 3.55 × 10/L and neutrophils 1.85 × 10/L were reexamined, and after discontinuation of leucogen tablets (June 2023), WBC 3.30 × 10/L and neutrophils 1.54 × 10/L were reexamined, followed by self-administration of compound donkey-hide gelatin slurry, WBC 4.16 × 10 ^/L and neutrophils 1.89 × 10 ^/L in July 2023, and after discontinuation of compound donkey-hide gelatin slurry (September 2023), WBC 3.10 × 10 ^/L and neutrophils 1.26 × 10 ^/L were again decreased.

**Figure 2:**
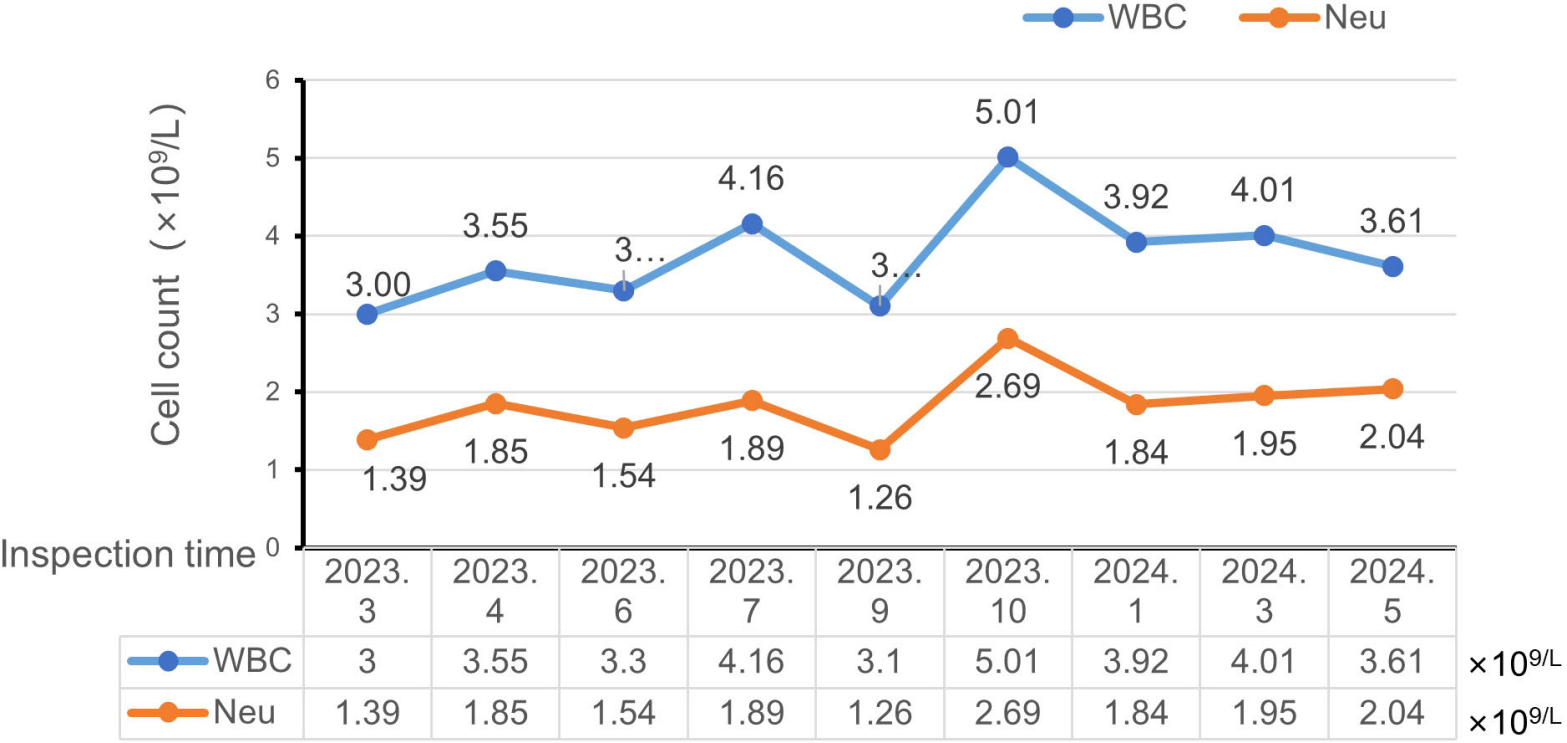
Temporal trends in leukocyte and neutrophil counts during tamoxifen therapy and post regimen adjustment. WBC, White blood cell; reference range: 3.5-9.5×10^9^/L; Neu, Neutrophils; reference range:1.8-6.3×10^9^/L.

In view of recurrent leukopenia and neutropenia, it was recommended to complete bone marrow aspiration to identify the presence or absence of hematologic diseases, and the patient refused bone marrow aspiration because he was afraid of trauma and believed that it could be improved after the application of drugs. According to the Naranjo adverse drug reaction probability scale, leukopenia and neutropenia were possibly related to tamoxifen (score 7), so tamoxifen was discontinued in October 2023. According to the 2023 Chinese guidelines for the diagnosis and treatment of breast cancer, the recommended regimen of OFS (leuprorelin acetate, 3.75 mg subcutaneously every 4 weeks) combined with aromatase inhibitors (exemestane 25 mg once daily) was switched. White blood cell and neutrophil counts returned to normal within 4 weeks and remained stable for 6 months. Currently, the patient continued to receive leuprolide combined with exemestane. Time trends in leukocyte and neutrophil counts are shown in **Figure 2**.

## Discussion

Tamoxifen-induced hematological toxicities are extremely rare, with less than 20 cases reported in the literature (4–9, 20, 21). The underlying mechanisms remain incompletely understood, but may involve immune-mediated destruction (type II hypersensitivity), direct myelosuppression, or disruption of estrogen receptor signaling in hematopoiesis.

According to the Gell-Coombs classification, a type II immune-mediated hypersensitivity reaction is considered a potential mechanism for agranulocytosis induced by certain drugs, including tamoxifen (6). Although specific antibodies were not detected in our case, the temporal relationship between drug exposure and cytopenias, relapse after accidental rechallenge (continued administration of compound donkey-hide gelatin), and rapid resolution after drug withdrawal strongly supports an immune-mediated mechanism. Two previous articles have reported bone marrow suppression associated with long term tamoxifen use (7, 8). One report described agranulocytosis co-occurring with fatal acute liver failure (9), supporting the potential risk of bone marrow suppression and/or immune-mediated hematological toxicity with tamoxifen. The Drug-induced Lymphocyte Stimulation Test (DLST) has high specificity (>93%) in identifying immune-mediated adverse reactions (10). Although DLST was not performed in this case, the rapid recovery after drug discontinuation closely resembles the case of tamoxifen-induced agranulocytosis reported by Juge (11), further supporting the involvement of an immune mechanism.

Tamoxifen’s molecular structure may also explain its rare hematological toxicity. The triphenylethylene core resembles compounds forming reactive metabolites capable of haptenation, potentially triggering immune responses (25). CYP2D6-mediated metabolism generates active metabolites (e.g., 4-hydroxytamoxifen) with enhanced receptor affinity and altered electronic properties (26).

The molecule’s conformational flexibility may allow interactions with hematopoietic proteins (27). Experimentally, tamoxifen inhibits GM-CSF-driven dendritic cell differentiation, likely by disrupting ERα-mediated cytokine signaling (12, 13). Its polarized regions (electronegative oxygen, protonatable amine) facilitate both specific receptor binding and potential off-target effects (28).

Future structure-activity studies could identify modifications retaining efficacy while reducing hematological risk.

Recent studies suggest that some patients may have underlying susceptibility factors, such as polymorphisms in drug-metabolizing enzymes (e.g., CYP2D6) or a predisposition to immune hypersensitivity (11). Estrogen receptor signaling is involved in maintaining immune homeostasis by regulating the differentiation fate of myeloid progenitors (Flt3 MP) in the bone marrow (12). ERα activation promotes myeloid differentiation in the context of GM-CSF – dominated inflammation; whereas ERα signaling instead suppresses cell survival in the Flt3 ligand-dominated homeostatic environment. As a selective estrogen receptor modulator (SERM), tamoxifen may disrupt granulocyte differentiation by interfering with ERα-mediated cytokine signaling crosstalk. This mechanism has been validated in experimental models: tamoxifen significantly inhibits GM-CSF driven dendritic cell differentiation (13). Successful switching to OFS combined with AI regimen in this setting is strongly associated with complete elimination of ER ligand interference in the bone marrow microenvironment.

Compound Ejiao Jiang, a traditional Chinese medicine preparation containing donkey-hide gelatin, red ginseng, codonopsis root, and prepared rehmannia root, is traditionally used to tonify both Qi and blood. Studies have reported its efficacy in improving chemotherapy-induced leukopenia, possibly through promoting hematopoietic growth factors (e.g., IL-3, IL-6, GM-CSF) and stimulating progenitor cell proliferation (14–18). In this case, transient increases in WBC and neutrophil counts were observed during its administration, but counts decreased again after discontinuation and failed to stabilize. This indicates that Compound Ejiao Jiang provided only temporary supportive effects and could not reverse tamoxifen-induced hematological toxicity, which ultimately required discontinuation of the causative drug.

Because of the rarity of this adverse reaction and potential confounding factors, including prior chemotherapy and the use of supportive medications (leucogen tablets, compound donkey-hide gelatin), the diagnosis of tamoxifen-induced neutropenia and leukopenia is challenging. However, exclusion of other common causes (infection, nutritional deficiencies, concomitant medications) and a clear temporal relationship between drug exposure and cytopenias, as well as prompt normalization following tamoxifen discontinuation, strongly supports this diagnosis. A score of 7 on the Naranjo scale further confirms possible adverse drug reactions. This report provides a detailed clinical timeline and highlights the importance of considering drug-induced cytopenias in breast cancer patients receiving endocrine therapy. However, limitations include lack of bone marrow aspirate to rule out myelodysplastic syndrome or bone marrow infiltration, and lack of confirmatory immunological or genetic testing. Nonetheless, the clinical course strongly supports this diagnosis.

The decision to switch to ovarian function suppression (OFS) plus aromatase inhibitor (AI) was based on recurrent leukopenia and neutropenia. Evidence from TEXT and SOFT trials further confirms this treatment option, demonstrating that OFS combined with AI provides meaningful absolute benefit over tamoxifen alone in premenopausal women with hormone receptor-positive early breast cancer, particularly in intermediate-risk patients. According to a combined analysis of TEXT and SOFT trials, in intermediate-risk patients, exemestane plus OFS prolonged the 5-year breast cancer-free interval by approximately 5% compared with tamoxifen alone (19). In addition, the 12-year results of the SOFT trial showed that both exemestan-OFS and tamoxifen-OFS achieved high survival rates (> 95%) for patients who did not receive chemotherapy, with only marginal differences, confirming that OFS – AI is a reasonable alternative for patients who cannot tolerate tamoxifen (3). In the present case, rapid normalization of blood counts after switching to leuprolide and exemestane confirmed the pathogenic effect of tamoxifen and illustrated the feasibility of this alternative strategy.

In addition, this case highlights the limited efficacy of supporting drugs such as compound donkey-hide gelatin lowering, which provide only transient improvement but are unable to reverse potential drug-induced toxicity (14–18). Ultimately, this report adds to the sparse literature on tamoxifen-induced hematological toxicities and highlights the need for vigilance and prompt intervention in patients receiving long-term endocrine therapy.

## Conclusion

This case highlights several important considerations for clinicians:

1. Although severe hematological toxicity due to tamoxifen is rare, vigilance is necessary during long-term administration.
2. Regular monitoring of complete blood count is recommended for patients on long-term tamoxifen therapy, especially during the initial treatment phase.
3. Persistent or progressive leukopenia/neutropenia should raise strong suspicion for an adverse drug reaction once other common causes—such as infection, nutritional deficiencies, or concomitant medications—have been excluded.
4. If tamoxifen is suspected, prompt discontinuation is essential; a drug-induced lymphocyte stimulation test (DLST) can be performed to confirm immune-mediated hypersensitivity (specificity >93%), and pharmacogenetic testing for metabolic enzymes (e.g., CYP2D6) may be considered to assess the risk of metabolic abnormalities.
5. For premenopausal patients requiring ongoing endocrine therapy, switching to an OFS plus AI regimen is a safe and effective alternative strategy.

## Data Availability

The raw data supporting the conclusions of this article will be made available by the authors, without undue reservation.
